# 1422. A Multi-Center Evaluation of Omadacycline for Multi-Drug Resistant Infections

**DOI:** 10.1093/ofid/ofac492.1251

**Published:** 2022-12-15

**Authors:** Amer El Ghali, Amer El Ghali, Sara Allosaimy, Taylor Morrisette, Kyle C Molina, Jeremy J Frens, Ryan W Stevens, Jeannette Bouchard, P Brandon Bookstaver, Michael P Veve, Raaga Vemula, Julie Philley, Nicholas S Rebold, Dana Holger, Ashlan J Kunz Coyne, Abdalhamid M Lagnf, Kristen Lucas, Paige Witucki, Michael J Rybak

**Affiliations:** Anti-infective Research Laboratory - Wayne State University, Detroit, Michigan; Anti-infective Research Laboratory - Wayne State University, Detroit, Michigan; Wayne State, Detroit, Michigan; Medical University of South Carolina College of Pharmacy, Charleston, South Carolina; University of Colorado Hospital, Aurora, Colorado; Cone Health, GREENSBORO, North Carolina; Mayo Clinic, Rochester, Minnesota; Prisma Health Richland - University of South Carolina, Columbia, South Carolina; Prisma Health Richland - University of South Carolina, Columbia, South Carolina; Eugene Applebaum College of Pharmacy and Health Sciences, Detroit, Michigan; University of Texas at Tyler, Tyler, Texas; University of Texas at Tyler, Tyler, Texas; Wayne State University, Detroit, Michigan; Wayne State University, Detroit, Michigan; Wayne State University, Detroit, Michigan; Wayne State University, Detroit, Michigan; Wayne State University, Detroit, Michigan; Wayne State University, Detroit, Michigan; Eugene Applebaum College of Pharmacy and Health Sciences, Detroit, Michigan

## Abstract

**Background:**

Multi-drug resistant (MDR) pathogens are a continuously evolving threat for which there are limited effective treatment modalities. Omadacycline (OMC) is a novel oral and intravenously administered aminomethylcycline with potent *in-vitro* activity against MDR gram-negative and gram-positive pathogens. Post-marketing data investigating its potential place in the antibiotic armamentarium is limited. This study aimed at describing the clinical success and tolerability of patients receiving OMC for various infections.

**Methods:**

This was a multicenter, retrospective, observational study conducted from January 2020 to March 30, 2022. We included all patients ≥ 18 years of age that received OMC for any infection not related to *Mycobacterium spp.* for ≥72 hours. The primary outcome was clinical success at 30 days, defined as survival, the absence of persistence or re-emergence of infection during or after therapy, and the absence of escalation or alteration of OMC. Incidence of adverse effects while on OMC and reasons for OMC utilization were also analyzed.

**Results:**

This analysis included 19 patients from 10 distinct academic centers. The median age was 50 years (IQR, 37-63), 58% were male, 63% were Caucasian, and the median APACHE II, Charlson and SOFA scores were 10 (IQR 5-12.5), 4 (IQR 2-6.3), and 1 (IQR 0 – 2.25), respectively. Most patients had a bone and joint (8/19, 42.1%) or intra-abdominal infections (4/19, 21.1%), all with positive cultures. Eight patients (44.1%) had a polymicrobial infection and 4 (22.2%) had bacteremia. The most common pathogens encountered were Carbapenem-resistant *A. baumannii* (5/19, 27.8%), *E. coli* (5/19, 27.8%), Vancomycin-resistant *Enterococcus* spp. (4/19, 22.2%), and *K. pneumoniae* (3/19, 16.7%). Clinical success was observed in 14 patients (74%) and 7 (36.8%) received combination therapy. Adverse drug reactions were reported in 3 patients (15.8%) and of those patients, none discontinued therapy. Reasons for prescribing OMC were mostly attributed to oral availability (12/14, 85.7%) and resistance to other agents (7/14, 50%).

**Conclusion:**

OMC demonstrated clinical effectiveness and safety against a large number of MDR pathogens. More robust, prospective, comparative studies are needed to confirm our preliminary findings.

**Disclosures:**

**P. Brandon Bookstaver, PharmD**, Spero Therapeutics: Advisor/Consultant.

